# Auditory Stimuli Mimicking Ambient Sounds Drive Temporal “Delta-Brushes” in Premature Infants

**DOI:** 10.1371/journal.pone.0079028

**Published:** 2013-11-11

**Authors:** Mathilde Chipaux, Matthew T. Colonnese, Audrey Mauguen, Laure Fellous, Mostafa Mokhtari, Oscar Lezcano, Mathieu Milh, Olivier Dulac, Catherine Chiron, Rustem Khazipov, Anna Kaminska

**Affiliations:** 1 Inserm, U663, Paris, France; University Paris-Descartes, Paris, France; 2 CEA, Neurospin, Gif-sur-Yvette, France; 3 Department of Pediatric Neurosurgery, Fondation Ophtalmologique A. de Rothschild, Paris, France; 4 Inserm, U901/Inmed, Aix-Marseille University, Marseille, France; 5 Department of Pharmacology and Physiology and Institute for Neuroscience, The George Washington University, Washington, D. C., United States of America; 6 AP-HP, Department of Neonatal Care Unit, Cochin-Saint Vincent de Paul Hospital, Paris, France; 7 AP-HP, Department of Neonatal Intensive Care Unit, Kremlin-Bicêtre Hospital, Kremlin-Bicêtre, France; 8 Laboratory of Neurobiology, Kazan Federal University, Kazan, Russia; 9 AP-HP, Department of Clinical Neurophysiology, Cochin-Saint Vincent de Paul Hospital, Paris, France; 10 AP-HP, Department of Clinical Neurophysiology, Necker-Enfants Malades Hospital, Paris, France; Hospital Nacional de Parapléjicos - SESCAM, Spain

## Abstract

In the premature infant, somatosensory and visual stimuli trigger an immature electroencephalographic (EEG) pattern, “delta-brushes,” in the corresponding sensory cortical areas. Whether auditory stimuli evoke delta-brushes in the premature auditory cortex has not been reported. Here, responses to auditory stimuli were studied in 46 premature infants without neurologic risk aged 31 to 38 postmenstrual weeks (PMW) during routine EEG recording. Stimuli consisted of either low-volume technogenic “clicks” near the background noise level of the neonatal care unit, or a human voice at conversational sound level. Stimuli were administrated pseudo-randomly during quiet and active sleep. In another protocol, the cortical response to a composite stimulus (“click” and voice) was manually triggered during EEG hypoactive periods of quiet sleep. Cortical responses were analyzed by event detection, power frequency analysis and stimulus locked averaging. Before 34 PMW, both voice and “click” stimuli evoked cortical responses with similar frequency-power topographic characteristics, namely a temporal negative slow-wave and rapid oscillations similar to spontaneous delta-brushes. Responses to composite stimuli also showed a maximal frequency-power increase in temporal areas before 35 PMW. From 34 PMW the topography of responses in quiet sleep was different for “click” and voice stimuli: responses to “clicks” became diffuse but responses to voice remained limited to temporal areas. After the age of 35 PMW auditory evoked delta-brushes progressively disappeared and were replaced by a low amplitude response in the same location. Our data show that auditory stimuli mimicking ambient sounds efficiently evoke delta-brushes in temporal areas in the premature infant before 35 PMW. Along with findings in other sensory modalities (visual and somatosensory), these findings suggest that sensory driven delta-brushes represent a ubiquitous feature of the human sensory cortex during fetal stages and provide a potential test of functional cortical maturation during fetal development.

## Introduction

Neuronal activity plays an important role in sculpting nascent circuits in the cerebral cortex at all stages of neuronal development, a process that has been studied primarily in sensory regions in rodents and monkeys [1]–[6]. Sensory cortex is organized in topographic maps each of which is tuned to input in a specific sensory modality. During early development, activity in sense organs is transmitted through the thalamus [7], [8] to the cortex [9]–[11] where it confirms or modifies thalamo-cortical connections established according to cell surface and diffuse guidance cues [8],[12]–[17]. During later development, sensory experience further refines and modifies these initial patterns of connectivity [18]–[22]. In rat, during the initial circuit formation in the first two post-natal weeks, electroencephalographic (EEG) activity in primary cortices is primarily driven by input from the sensory periphery which drives in the corresponding cortical areas characteristic EEG pattern of “spindle-bursts” consisting of burst of rapid oscillations nested in a slow delta-wave [9], [10],[23]–[26]. The frequency, size and duration of these events are variable, and are at least partially determined by the characteristics of activity at the sensory periphery [27]. For example, brief stimulation of single whiskers causes high-frequency (gamma band) oscillation bursts of very restricted topography allowing spatio-temporal thalamo-cortical binding [8], [28]. By contrast, retinal waves, which are of long-duration and synchronize large areas of the receptor sheet, generate long-duration waves of slower frequency in the visual cortex [11]. The role of early activity is particularly important during so-called “critical” periods when synaptic plasticity is enhanced [6], [29]. During the critical period, alteration of sensory stimuli (in terms of type and amount) results in formation of aberrant circuits and may lead to irreversible functional deficits. For example, in rodents, the formation of tonotopic maps is specifically influenced by early acoustic environment: exposure to pure tones results in accelerated emergence and over-representation of those specific tone frequencies in the auditory cortical map [30]. In primates, cortical sensory maps develop *in utero* so that at birth the newborn already displays remarkable capacities for recognition of sensory inputs for different modalities [1], [31], [32]. One approach to study cerebral activity during the fetal period is to record cortical activity in preterm infants. In the absence of cerebral injury, the maturation of EEG patterns takes place “*ex utero*” in comparable manner to that of fetuses “*in utero*” [33], [34]. EEG in premature infant displays unique activity patterns among which delta-brush is predominant [33], [34]. Delta-brushes are high amplitude slow waves (50–300 µV) superimposed with rapid (>8 Hz) oscillations, that are expressed in all cortical areas including sensory regions beginning around 28 postmenstrual weeks (PMW) and fading near term [34]. As key EEG graphoelements of this period, delta-brushes serve as a clinical marker to estimate brain maturity and health during neurophysiologic examination [34].

For a long time, delta-brushes have been considered as an internally generated spontaneous activity pattern. However, more recent studies revealed tight link between delta-brushes and sensory inputs. Indeed, it has been shown that somatosensory and visual stimuli reliably evoke delta-brushes respectively in central and occipital cortical areas [35]–[38]. Along with the disappearance of delta-brushes and a transition from discontinuous to continuous temporal organization of activity, visual and somatosensory-evoked delta-brushes are also eliminated around 35 PMW, switching to a mature-like short duration, low amplitude response [38]. Similar findings in somatosensory and visual systems raise the hypothesis that such a paradigm also operates during development of other sensory systems. Among different sensory modalities, the auditory system is of particular interest for several reasons: first extra-uterine visual and somatosensory signals barely pass the uterine wall while auditory signals reach the fetus with less attenuation; second, several observations indicate that auditory functions develop already *in utero*: indeed, human newborn can recognize the mother’s voice from prenatal exposure, is able to discriminate between different sounds (speech, music, combination of pure tones), and can remember acoustic cues of a target passage of a story read prenatally [39]–[44]. Such skills require early prenatal development of auditory function. Hearing begins at the end of the second trimester of gestation when mechanical functioning of the cochlea matures and exposes the fetus to maternal sounds and attenuated auditory input from the external world [45], [46]. Behavioral responses and cortical auditory evoked potential (CAEP) to tone bursts can be evoked from 28 weeks of gestation, even before cochlear maturation is completed [42], [47]–[49]. Fetal behavioral responses are first observed to low frequencies. At 27 weeks of gestation, 96% of fetuses respond to 250 and 500 Hz tones but not to 1000 and 3000 Hz tones. Because the fundamental frequency of speech is around 225 Hz for females and 128 Hz for males, the human voice should constitute a salient auditory stimulus for the fetus, promoting language acquisition and attachment [50]. The ability for premature infants to discriminate between phonemes and between male and female human voices from 28 PMW has been recently demonstrated using functional optical imaging [51]. However, electrical cortical activity patterns associated with early auditory signal processing remain largely unknown.

In this study, we presented auditory stimuli mimicking two typical ambient sounds occurring in the neonatal ward: machine generated clicks and human voice, to test the hypothesis that environmental sounds trigger delta-brushes in the auditory cortex of premature infants, and to determine whether there is any EEG response selectivity for these two types of stimuli. Understanding the selectivity of early cortical responses to sounds has particular relevance for preterm infants, who are exposed to surfeit of technogenic sounds [52]. In addition evoking delta-brushes by sensory stimuli may provide a means for clinical testing of cortical functional development in humans during the fetal period.

## Methods

### 1/Participants

All premature infants referred to our neurophysiology department from October 2007 to June 2009 for EEG were screened for inclusion. Exclusion criteria were: intrauterine growth retardation defined as birth weight under the 10^th^ percentile for the gestational age; ventilation support; brain abnormality on ultrasound scan (excluding intraventricular hemorrhage grade I); history of perinatal asphyxia; abnormal acoustic oto-emissions, dysmorphia or congenital abnormalities; significant patent ductus arteriosus; clinical evidence of sepsis; documented congenital infection (e.g. cytomegalovirus), postnatal treatment with corticosteroids excluding hydrocortisone; abnormal neurodevelopment at any time during follow-up; and any condition that in the opinion of the investigator could alter brain development.

Forty-six premature infants were investigated in the department of neonatology comprising the neonatal intensive care unit (NICU) of Saint-Vincent de Paul Hospital (Paris, France), an academic center with regional maternity ward. This center was a level III center (defined in France by its ability to provide mechanical ventilation). Gestational age at birth was 29–30 (n = 12), 31–32 (n = 22), 33–34 (n = 11) and 35 (n = 1) weeks of gestation, age at recording was expressed as PMW (weeks of gestational age at birth+post-natal weeks) [53]. EEGs were performed as part of routine neurological follow-up according to the recommendations for premature infants [34]. Written informed consent was obtained from all parents. The clinical trial was in accordance with the Ethics Code of the World Medical Association, approved by our institutional review board, INSERM’s (French National Institute of Health and Medical Research) Ethics Committee, and was registered as a Clinical Research Study classified within “methodology of reference” under the n° 558/BB/PA/2005-12 (available on the ANR (French Research Agency) site under the n° ANR-05-NEUR-014-02). Clinical follow-up was over 12 months for 37 children, between 2 and 12 months for 8. One infant was lost to follow-up. Psychomotor outcome was considered normal, and all children who had reached 18 months or more were able to walk normally.

### 2/EEG Recording

Forty-nine of fifty recordings were analyzable. Four premature infants had two consecutive recordings. Recording included cardiogram and respiration, and was performed according to the 10/20 international system, using 9 electrodes for the first 19 recordings and 11 for the later recordings (adding 2 temporal posterior electrodes). The medial frontal polar (FPZ) electrode served as reference. Ground was placed on the mastoid. EEG sessions lasted at least 45 minutes to record quiet and active sleep. Since the fetus spends an estimated 80% time sleeping (each cycle lasting from 40 minutes at 27–30 PMW to 70 minutes at term, comprising active sleep (AS), and quiet sleep (QS) and awake state), we limited our study to QS and AS, the most behaviorally relevant vigilance states [54]. Signals were amplified (1000 x), band pass filtered at 0.01–97 Hz and digitized at 256 Hz, using the Deltamed Coherence EEG system. Offline analysis was made with the Coherence review program (Deltamed/Natus Paris, France) and Matlab (Mathworks, Natick MA).

Auditory stimuli were presented with constant sound volume threshold via a loud-speaker placed 10 cm behind the head. Sound intensity (sound pressure level using the hearing threshold pressure of 2×10^−6^ Pa as baseline) was measured during the entire EEG recording with a sonometer (Chauvin Arnoux, C.A 832) placed near the head. The direct current (DC) output of the sonometer was connected to a DC input of the amplifier, allowing insertion of stimulation markers in the EEG recording using threshold detection. The background noise level in the neonatal department was between 50 and 53 dB Sound Pressure Level (SPL). Infants were simultaneously video-monitored.

Two stimulus protocols were used in this study. For the first 32 recordings (“triggered population”), in order to better separate the response from background activity, stimuli were triggered manually during periods of EEG hypoactivity in QS. We applied a minimum interval of 10 seconds between stimuli. The stimulus consisted of a recorded male voice pronouncing the word “bébé” (meaning “baby” in French) recorded in a.wav windows file (lasting 420 ms, with a peak intensity of around 70–75 dB (SPL), and a frequency peak at 400 Hz). Stimuli were calibrated in order to prevent the child from awakening. Post-experiment analysis revealed that this stimulus also contained a low intensity 4 ms-long “click” that slightly exceeded the background noise level of the neonatal department (with a peak intensity of 56–62 dB (SPL) and predominant frequency around 100 Hz) which corresponded to the opening of the.wav file and preceded the voice stimulus by 400 ms. Despite the fact that the investigator could not hear the click during the experiment, some infants exhibited electrographic responses to it.

For the subsequent 17 recordings, each stimulus (“click” and voice) was presented separately with the same intensity as for the triggered protocol, using a pseudo-random design every 20 second to avoid habituation, in both QS and AS (“periodic population”). This allowed us to determine the effect of the stimulus type and the sleep stage, and to eliminate the potential confounding effect of stimulating only during periods of EEG hypoactivity of QS.

### 3/EEG Signal Analysis

**Figure 1:**
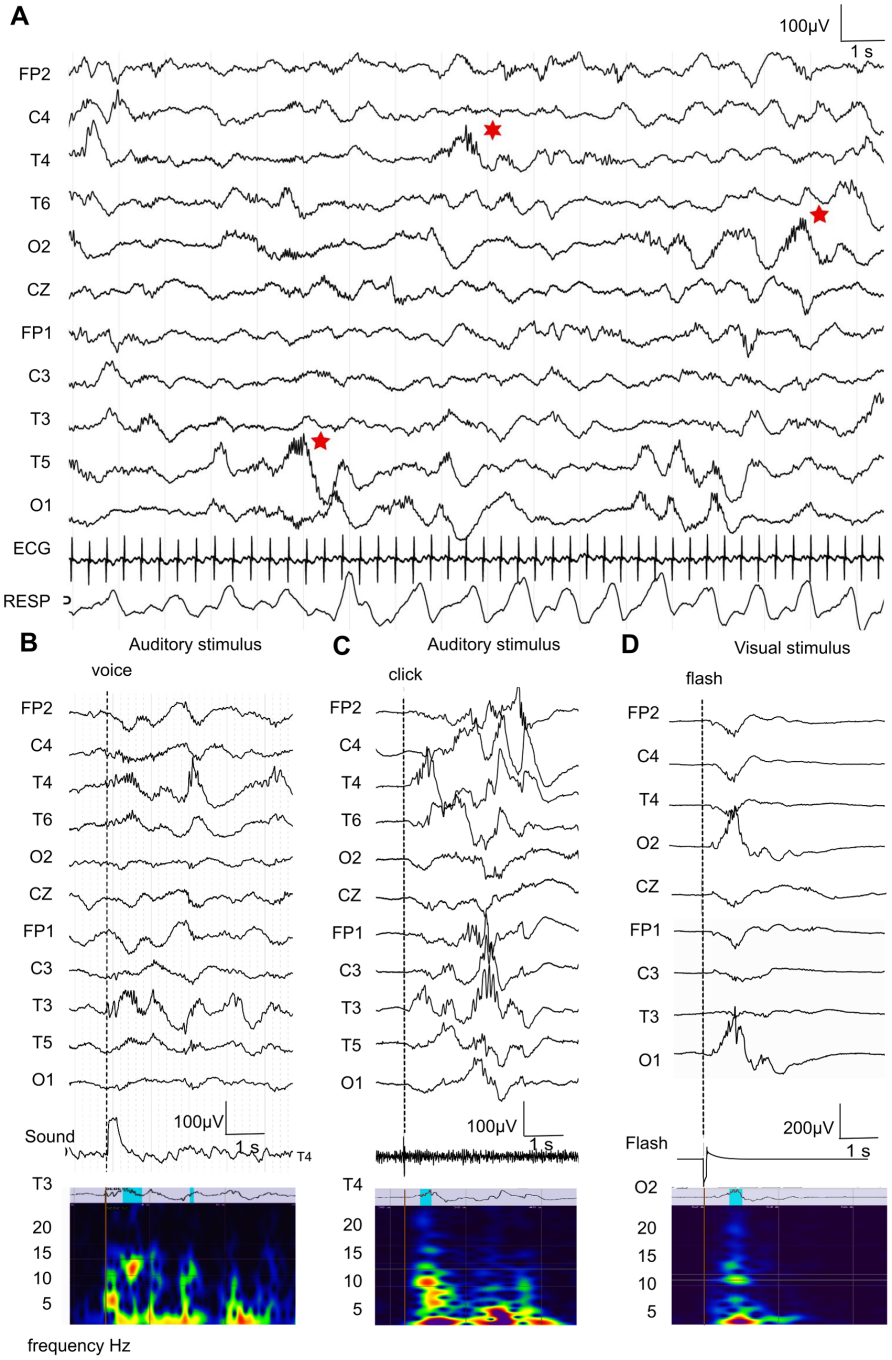
Auditory evoked delta-brushes by “click” and voice in temporal cortex. **A.** Electroencephalogram (EEG) in a 31 postmenstrual week (PMW) premature infant during active sleep (AS) showing continuous activity with diffuse slow waves and multifocal delta-brushes (red stars). Traces are shown with mean reference montage (high-pass filter 0.53 Hz, notch filter 50 Hz, electrodes placement using 10/20 international system). ECG: electrocardiogram, RESP: respiration, **B.** Example of temporal delta-brushes evoked by single auditory voice stimulus, recorded during quiet sleep (QS) at 31 PMW. Corresponding wavelet analyses of T3-reference derivation is shown below (delta-brushes are outlined in blue on the T3-reference trace above). **C.** Example of temporal delta-brushes evoked by single auditory “click” stimulus recorded during QS at 31 PMW and corresponding wavelet analyses on T4-reference derivation. **D.** Representative example of occipital delta-brushes evoked by single visual flash stimulus, recorded during QS at 32 PMW with corresponding wavelet analysis on O2-reference derivation.

The EEG was first analyzed by a trained neurophysiologist in order to confirm normality and adequacy for gestational age. Artifact free periods of QS and AS were selected for further analysis. QS was determined by the presence of discontinuous, semi-discontinuous or “tracé alternant” EEG patterns (as appropriate for age), regular respiration and cardiac rhythm, and absence of phasic movements. AS was determined by the presence of continuous EEG activity, irregular respiration and the presence of phasic movements [34]. EEG was visually analyzed using mean reference and a conventional 0.3 second time constant (0.53 Hz high pass filter) (Fig. 1 A).

We applied three different EEG signal analyses to quantify developmental changes in spontaneous and auditory-evoked responses: i) automatic detection and quantification of delta-brushes based on their fast component to test if the occurrence of delta-brushes was increased after stimulation, ii) multi-taper spectrogram analysis and averaging of fast and slow components to test if the mean auditory responses were consistent with evoked delta-brushes, iii) total power spectral analysis of evoked response to determine their frequency characteristics without prior assumptions. Since EEG patterns in the premature infant undergo fast maturational changes (every 1–2 weeks) and visually evoked delta-brushes are known to decrease after 35 PMW [38], we analyzed expected maturational changes in auditory evoked responses week by week (for Multi-taper spectrogram analysis) and dividing all the recordings into 3 PMW age groups (before 34 weeks, 34–35, and from 36 to full term) for the 3 other analyses.

#### Automatic detection and quantification of the fast component of delta-brushes

Delta-brushes comprise a slow wave superimposed with rapid oscillation “brushes” that rarely occur in isolation, thus we based delta-brush detection on their rapid component. Wavelet time-frequency (Deltamed Coherence software) was used to detect “brushes” with a frequency band between 8 and 20 Hz, lasting from 0.2 to 3 seconds, with a minimal inter-event duration of 0.3 seconds. Detection was calibrated for each infant using a reference period lasting 20 seconds showing one or more delta-brushes. A reference level for the integrated frequency power (8–20 Hz) was calculated based on the “brush” with the highest amplitude on that period. A threshold for brush detection at most of 20% of the reference level and at least 20 µV of amplitude above the baseline was determined for each infant. After detection and visual verification of “brushes”, the frequency of their occurrence was determined within the entire artifact-free recording in QS and AS for the baseline and as evoked “brushes” within 2 seconds following each stimulus. A ratio of evoked/spontaneous “brushes” frequency was calculated separately for QS and AS in the “periodic population” for each electrode, without distinction for the stimulus type.

#### Multi-taper spectrogram analysis and averaging of the fast and slow components of the auditory evoked response

We characterized the mean of auditory evoked responses in each recording in both stimulus groups by averaging both the amplitude of evoked responses and the power of rapid oscillations. The frequency of the evoked responses was determined by multi-taper analysis of a one second moving window (100 ms steps, 2 tapers, 1–50 Hz) using Chronux [55]. We compared the two seconds following the stimulation to the 10 seconds preceding it (used as baseline). Duration of the rapid oscillations was calculated as the number of windows showing a significant increase in 8–20 Hz power over the baseline (>95% confidence interval). The characteristics of the slow component (delta) of the evoked response were calculated from the mean auditory evoked response at temporal electrodes. Two measures were used: the total charge flux was calculated as the sum of EEG within two seconds following stimulation, while the maximum amplitude was the peak of the negative wave of the average response. All electrodes were examined versus the mean reference. Topographic mapping was performed on the mean responses for 31–34 PMW. The peak response was chosen for the time point mapped, and the value at that time point was converted into a pseudo color map using the EEGLAB software package [56].

#### Total power spectrum analysis according to age and stimulus type

In order to analyze the whole spectral power separately by age, stimulus type, sleep stage and electrode, the power spectra for the two second windows immediately preceding and following each stimulus were compared using the Fast Fourier Transform (FFT) algorithm with 0.5 Hz frequency resolution (Deltamed Coherence FFT analysis). This comparison was made in both “periodic” and “triggered” populations. Power spectra were broken into five EEG frequency bands (delta: 0.5–3.5 Hz, theta: 4–7.5 Hz, alpha: 8–13 Hz, beta: 13.5–31.5 Hz, global: 0.5–31.5 Hz). To compare cortical responses to auditory stimuli to those following other sensory stimuli, and to exclude responses related to vigilance changes, we performed the same analyses on data drawn from 20 previously recorded premature infants who had received 100 ms light flashes during EEG hypoactivity in QS [38].

### 4/Statistics

For delta-brush quantification analysis multivariate analysis of variance (MANOVA) and Tukey post-hoc tests were performed considering electrode, side and PMW age groups as independent factors; distributions were considered to meet the criteria for normality if n>10 for each group and the Shapiro-Wilk test for normality failed to reject the null hypothesis (p<0.05). To determine if the occurrence or power of the evoked “brushes” were elevated significantly over the baseline, the mean fold increase for each electrode and age range was subjected to a one sample t-test in order to reject the null hypothesis that ratio = 1 (i.e. no elevation over the baseline). We considered alpha level significant when p<0.05 after Bonferroni correction for multiple comparison tests. Analysis of week-by-week changes in slow waves and rapid oscillations at temporal electrodes used the Kruskal-Wallis test of variance as a result of small n's at multiple ages. Tukey HSD post-hoc test was used to determine differences between ages. For the total power spectrum analysis we used hierarchical models with an unstructured correlation matrix, taking into account the correlation in multiple measures for a given premature infant. In order to ensure the normality of the data and apply models, the logarithm of the power spectrum measures was used in the analyses. The power spectrum was described as a ratio of the value following the stimulation compared to the baseline (multiplicative coefficient, not the logarithm) [57]. The validity of the models was checked through the normality of the residuals, using the Skewness and Kurtosis tests, and the values between −1.5 and +1.5 were considered as normal. Considering the high number of tests performed, results were considered significant when the p-value was inferior or equal to 0.01.

## Results

The numbers of recordings and stimuli protocols according to age are summarized in Table 1. In both (periodic and triggered) groups the spatio-temporal organization of EEG patterns was consistent with reported findings (Fig. 1A) [34], [58], [59].

**Table 1:** Characteristics of the population including infants’ age expressed in postmenstrual weeks (PMW), number (n) of recordings and type and number of sensory stimuli.

Auditorystimuli	Age(PMW)	<34	34–35	>35	Total
Periodicpopulation	EEGrecordings (n)	4	4	9	17
	PMW	32–33	34–35	36–37	32–37
	Click (n)	130	175	435	740
	Voice (n)	167	148	413	728
Triggeredpopulation	EEGrecordings (n)	11	9	12	32
	PMW	31–33	34–35	36–38	31–38
	Composite	250	173	194	617
Visualstimuli	EEGrecordings (n)	9	11		20
	PMW	31–33	34–38		31–38
	Flash (n)	147	253		390

Most recordings in the youngest (31–33 PMW) premature infants showed visually identifiable temporal delta-brushes in response to single auditory stimuli, either voice or “click”. Such responses were especially prominent in QS (Fig. 1B–C). Auditory evoked delta-brushes had a topography different from visually evoked delta-brushes, which were located in the occipital area (Fig. 1D).

**Figure 2:**
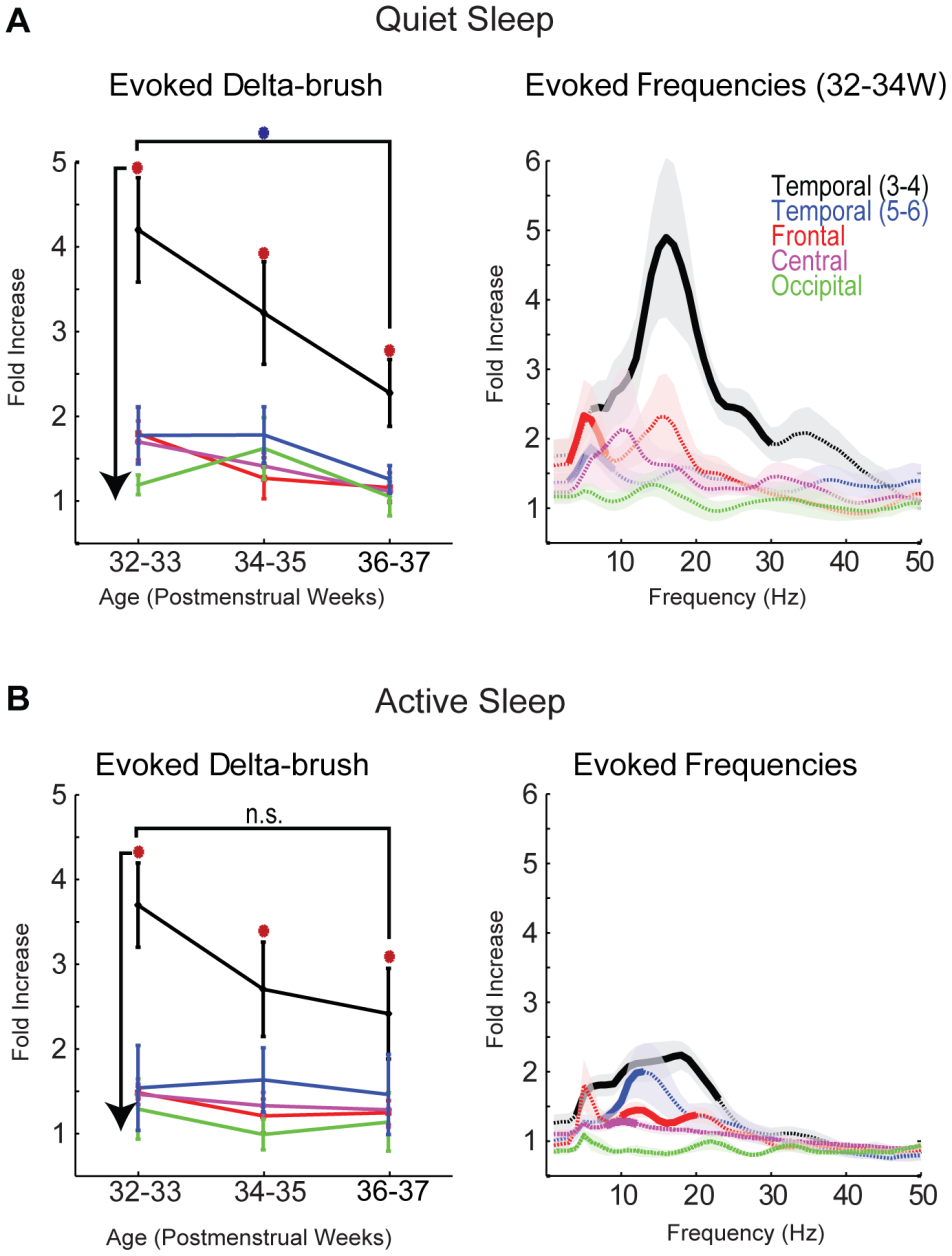
Age and sleep stage dependence of auditory evoked delta-brushes. **A–B: Left column:** Increased delta-brush occurrence (expressed as a ratio of evoked to spontaneous brushes) for the 2 second period following “periodic” auditory stimulation (“click” or voice)/baseline rate. QS and AS are graphed separately. Left and right electrodes are pooled and each electrode pair is shown by different colors (error bar is SEM). Orange circles show significant elevation above baseline (p<0.05), while the blue asterisk shows significant difference between age groups. **Right column:** elevation of frequency power (1–50 Hz) for 32–34 postmenstrual weeks. QS and AS are graphed separately Colored line denotes mean elevation for each electrode, while SEM is indicated by shaded region. Solid bold lines show the frequencies significantly elevated above baseline (p<0.05); dotted lines show non-significant frequencies.

Quantification of evoked “brushes” showed a significant effect of recording electrode, sleep stage and age group, but no effect of side (data not shown; MANOVA analysis p>0.05), thus further analyses of evoked responses combined both paired contralateral electrodes. Auditory stimulation increased the ratio of evoked to spontaneous “brush” occurrence only on mid temporal electrodes (T3, T4). This effect was observed for all age groups and both sleep stages (Fig. 2A–B left column). The peak response was observed at 32–33 PMW in QS (4.2±0.6 SEM). The ratio decreased with age, reaching a value of 2.3±0.4 at 36–37 PMW. MANOVA revealed an effect of recording site (p<0.0001) and age (p<0.0001) on the occurrence of evoked “brushes”. Interaction between both factors approached significance (p = 0.06). Post-hoc analysis revealed that these effects were due to a significant decrease in the response specifically on T3 and T4 electrodes between 32–33 PMW and 36–37 PMW age groups (p<0.05). During AS auditory stimulation also caused an increase in the frequency of evoked “brushes” only at T3 and T4 electrodes. The occurrence ratio decreased with age, but the effect was not significant (Fig. 2B left column). Thus auditory stimulation resulted in an elevated occurrence of “brushes” that was specific to mid temporal electrodes and more powerful in young preterm infants.

The multi-taper frequency-power characteristics of the evoked “brushes” at the age of their maximal occurrence (32–34 PMW) showed significant elevation specifically between 8 and 30 Hz that was limited to mid temporal electrodes (T3 and T4) in QS (p<0.05 for each frequency in 1 Hz increments) (Fig. 2A right column). During AS the auditory-evoked “brushes” were less elevated by stimulation and had slightly slower frequencies (5–25 Hz), but they remained restricted to the mid temporal recording sites (Fig. 2B right column).

**Figure 3:**
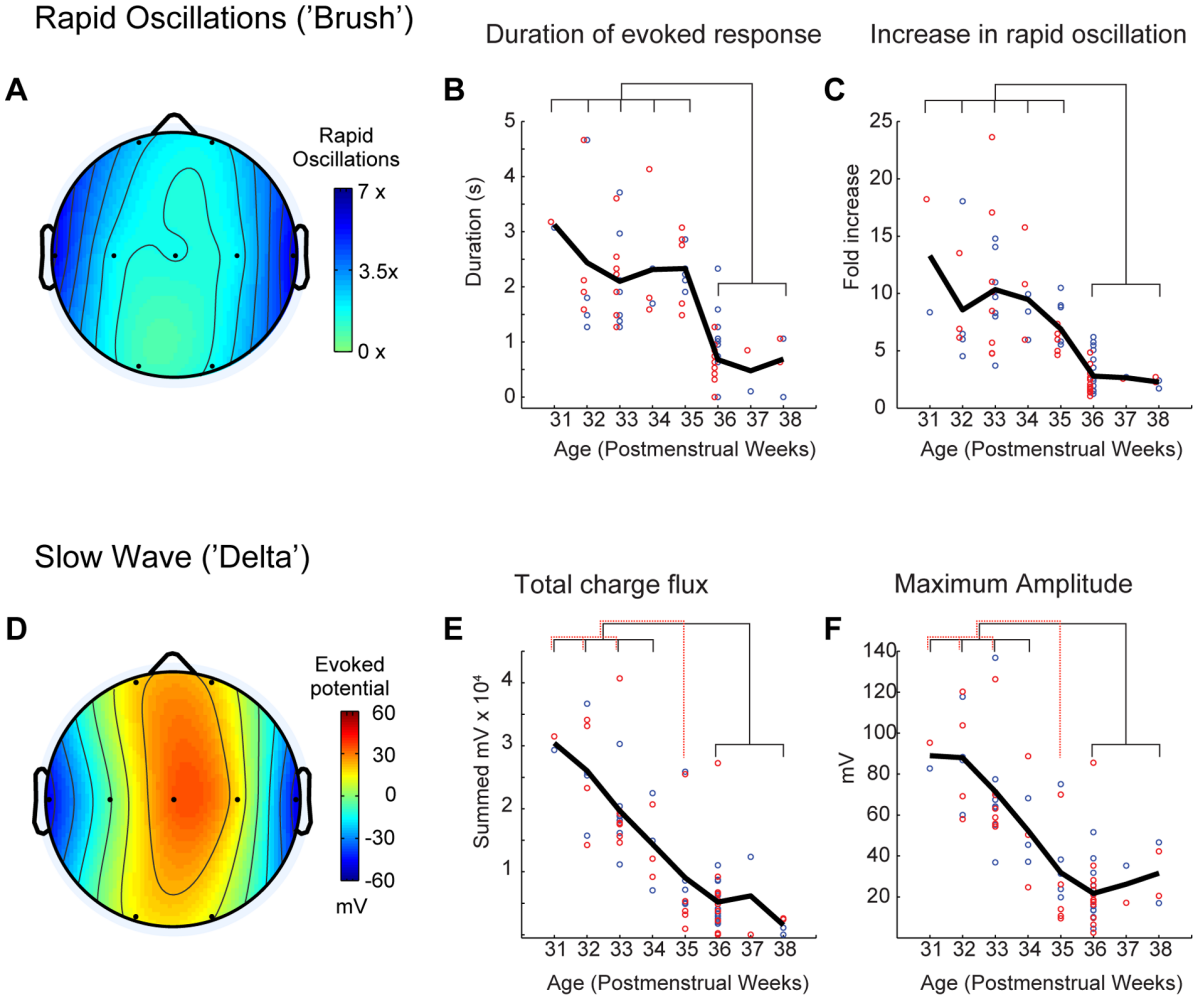
Developmental profile of auditory evoked responses. Pooled averages from all premature infants quantifying developmental changes in the rapid oscillations of the delta-brush (‘brush’) (A–C) as well as the slow negative wave (‘delta’) (D–F). **A.** Topography of rapid oscillations (8–25 Hz) evoked by auditory stimulation at 31–34 postmenstrual weeks (PMW) shows a temporal dominance. Frequency power is summed in the relevant frequencies 2 s after stimulation and compared to before. **B.** The duration of evoked rapid oscillations is shown for the T3 (red) and T4 (blue) electrode for each premature according to PMW. Thick black line shows population mean. Thin lines show significant difference between age groups (p<0.05). **C.** As B but peak increase in frequency power is plotted. **D.** Topography of the mean evoked potential at the peak of the negative potential (860 ms) after stimulus for 31–34 PMW. Blue color shows temporal prominence of negative potential. **E–F.** Quantification of evoked negative slow wave for T3 (red) and T4 (blue) electrodes. Thick black line shows population mean. Thin lines show significant difference between age groups (p<0.05). The summed negative potential in the 2 s following stimulation (‘Total charge flux’, E) and the peak amplitude of the negative potential (‘Maximum Amplitude’, F) are also shown.

The analysis of each component in the evoked responses for each infant by PMW (week by week) revealed a slight divergence in their developmental pattern. Analysis of the rapid oscillations (“brushes”), quantified as mean frequency power between 8 and 30 Hz, for each premature infant and each electrode during QS demonstrated a clear specificity for the mid temporal electrodes (T3 and T4), with little involvement even of the posterior temporal (T5 and T6) or central electrodes (C3 and C4) surrounding them (Fig. 3A). Both the duration of the evoked rapid oscillations and the fold increase in frequency-power remained high from 31 to 35 PMW, and then decreased rapidly by 36 PMW. Analysis of variance showed an effect of PMW for both duration (p<0.0001) and power increase (p<0.0001) (Fig. 3B–C). For both these parameters post-hoc analysis showed no significant differences between 31 and 35 PMW nor between 36 and 38 PMW; by contrast 31–35 PMW were different from 36 and 38 PMW. The slow component was also maximal on temporal electrodes but, in contrast to the rapid component, it underwent more gradual decrease in amplitude between 31 and 38 PMW (Fig. 3D–F). Analysis of variance showed an effect of age for both the total charge flux (p<0.0001) and the maximal amplitude (p<0.0001) (Fig. 3E–F). Post-hoc analysis of these measures showed three age groups, 31–34, 35 and 36–38 PMW, which differed significantly from each other (p<0.05) suggesting a gradual disappearance of the slow negative wave.

**Figure 4:**
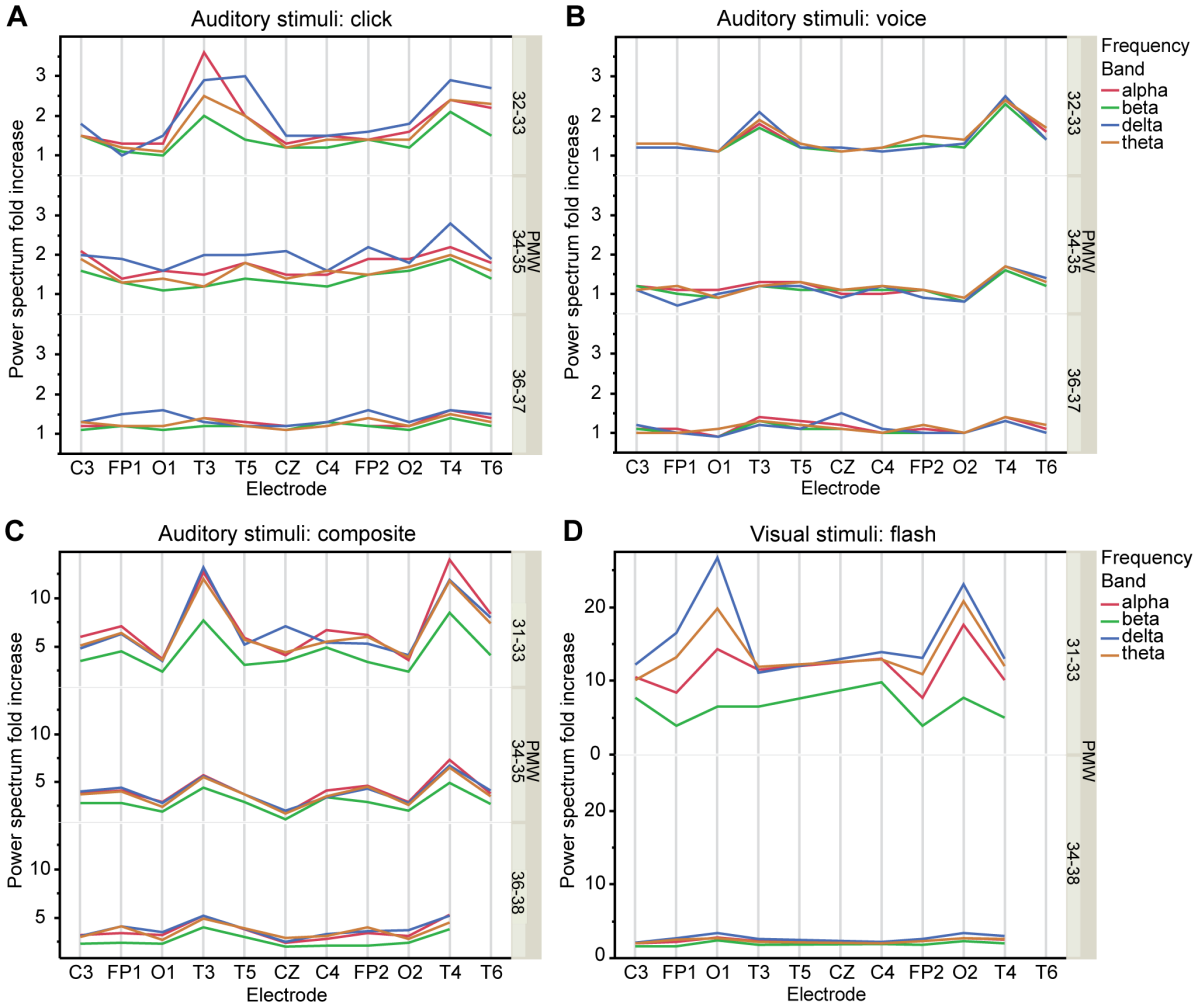
Power spectrum changes after auditory stimuli (click, voice and composite) and visual stimuli (flash) in quiet sleep according to age and EEG frequency bands. AB. Power spectrum increase after auditory stimuli “click” (A) and voice (B), for each electrode and in each frequency band. Graphs are shown for 3 age groups in postmenstrual weeks (PMW): <34 (above), 34–35 (middle) and >35 (below). At 32–33 PMW, click induces evoked responses on T3, T4, T5, T6, CZ electrodes whereas voice induces a response only on T4 and T3 (p<0.01) but the difference is not significant, (interaction test, all p-values >0.01). At 34–35 PMW, all electrodes record a response to “click” (p<0.01), but voice induces a response only on T4 (p<0.01) (interaction test, p<0.01). At 36–37 PMW, “click” induces a response at FP1, FP2, C4, O1, T4, T6 electrodes but voice induces a response only on T4 (p<0.01)**. C.** Power spectrum increase after auditory composite stimuli in “triggered” population. The increase is statistically significant on all electrodes in all age groups (p<0. 01). At 31–33 PMW the response is greater on mid temporal T3 and T4 electrodes than on the other electrodes. **D.** Power spectrum increase after visual stimuli. Visual stimulation evoke maximal occipital power spectrum increase at 31–33 PMW (p<0.01).

Standard Fourier-transform spectral analysis showed that baseline power was elevated for all electrodes in all frequency bands at 32–33 PMW relative to 34–37 PMW (p<0.001) (Table S1). Logarithmic transformations of power measures followed the normal law for all electrodes and all bands, in both age groups (Skewness indicator ranged between −0.8 and +0.7 and the values of the Kurtosis indicator ranged between −0.9 and +1.3). The maximal auditory responses were observed at mid temporal electrodes at 32–33 PMW in QS, both for “click” and voice stimuli (p<0.01); (Fig. 4A–B). “Click” stimulus induced also a response at temporal posterior and at vertex electrodes (p<0.01). However, responses to verbal and non-verbal stimuli could not be statistically differentiated at these ages (Tables S2, S3). From 34 PMW, in QS, the “click”-evoked response was present at most electrodes, whereas voice stimuli induced only a right temporal response (Fig. 4A–B); (Tables S4, S5, S6, S7). This difference between electrodes reached statistical significance in QS in the 34–35 PMW age group (p<0.01); (Table S4). The fold increase in frequency power on temporal electrodes induced by “click” dropped from 3.6 at 32–33 PMW to 1.6 at 36–37 PMW, and from 2.5 to 1.4 for voice (Tables S2, S3, S4, S5, S6, and S7).

The power spectral analysis of the evoked responses in the “triggered” population showed that for all frequencies all electrodes recorded a significant response to the composite stimulus (p<0.01). At 31–33 PMW the response was greater on mid temporal (T3 and T4) electrodes than in the other areas (14 fold increase) (Fig. 4C). By 36–38 PMW this temporal predominance had disappeared and a more diffuse and lower amplitude cortical response had developed (Fig. 4C). Visual stimulus induced maximal power spectra increase at occipital (O1 and O2) electrodes, this response was higher at 31–33 PMW than at 34–38 PMW (27 fold increase versus 3.5) (Fig. 4D).

## Discussion

Using standard EEG recording and stimuli mimicking ambient sounds, we observed auditory responses that were maximal on temporal areas before 35 PMW and consisted of slow waves mixed with rapid oscillations. By multiple quantitative measures these responses were identical to the commonly observed spontaneous activity pattern of “delta-brushes". Auditory evoked delta-brushes were present in both sleep stages and occurred in response to both verbal and non-verbal stimuli. We can exclude that they arise in relation to a non-specific, stimulus evoked, change in vigilance since similar though differently localized responses have also been obtained in the occipital cortex following light flash stimulation [38] and in the central cortex following somatosensory stimulation [35].

We suggest that our findings, as well as previous studies of preterm EEG [35]–[38], are relevant to normal foetal development, and are not due to prematurity-induced pathology since our volunteers met restrictive inclusion criteria which excluded abnormal neurodevelopment at any time during follow-up. Taken together with findings in other sensory modalities (visual and somatosensory), our results also suggest that delta-brushes driven by sensory periphery are a ubiquitous feature of the human sensorial cortex during fetal stages of development and likely contribute to the development of sensory maps. Studies in neonatal rat suggest that evoking delta-brushes by sensory stimuli can provide a test of functional maturation of the sensory cortex, particularly the integrity of the thalamo-cortical network. Indeed, lesion of relay thalamic nuclei has been shown to completely eliminate both sensory-evoked and spontaneous oscillatory bursts in cortex of neonatal rats [28].

Up to now, cortical auditory evoked responses in the premature infant have been performed using conventional techniques of CAEP, i.e. averaging hundreds of responses evoked by loud (80 dB hearing level) clicks, use of bipolar midline (but not temporal) electrodes, mastoid reference, and a 1–70 Hz band pass filter [60]–[62]. Between 28 and 34 PMW the major component of CAEP was described as a negative wave with a latency of 180–270 ms and postero-lateral maximal amplitude (C3′, C4′) [60] or a “premature negativity” on central and temporal areas with a latency of 200–250 ms [60]–[62]. Comparison of these data to our findings is limited since we used different stimuli and recording techniques. However our data revealed significant new conception of the early cortical auditory responses because important components are missed by standard techniques. These include (a) a rapid oscillatory component masked by trial averaging, (b) a negative response maximal at external temporal cortex which is not observed using midline electrodes, and (c) a very long latency to peak and high amplitude negative response that might be obscured by high-pass filtering. We therefore propose the following additional protocol for the CAEP examination of patients before 36 PMW: (i) analyze the rapid component by averaging the frequency power of individual responses; (ii) analyze responses at temporal electrodes; and (iii) minimize high-pass filtering.

We show that from 35 PMW on, there is a rapid elimination of temporal auditory-evoked delta-brushes. These changes are consistent with previous studies showing that CAEP changes in morphology, scalp topography, amplitude and latency between 36 to 40 PMW [45]. Before 36 PMW, CAEP occurs with a long delay (up to 1000 ms) and is predominantly negative across the scalp with maximal amplitude reached in postero-lateral regions. This is opposite in polarity to the CAEP recorded in full-term newborn, when the typical waveform recorded at the midline consists mainly of a broad positive peak, P2 [45], [60]–[62]. While rapid growth and cortical sulcation could explain the inversion of the CAEP dipole originating from auditory cortex, it was also hypothesized that the development of intra-cortical synaptic connections concomitant with a rapid increase in synaptic density may explain the changes of morphology and topography of the CAEP [45], [63], [64]. Findings in the visual cortex of premature infants and neonatal rats suggest that the modification in auditory responses is due to changes in cortical processing of sensory input. In premature infants before 34 PMW light flashes evoke delta-brushes on occipital electrodes, but after 36 PMW delta-brushes are replaced by small but mature-like visual evoked potentials (VEP) [38]. In the neonatal rat, in which cortical responses to light flashes and retinal activity were recorded in the visual cortex with depth EEG, evoked “spindle-bursts” were present in all cortical layers amplifying the sensory input and enabling a primitive form of vision. This period of burst-based amplification of sensory input was called the “bursting period”. The switch from the bursting period to mature and localized VEP occurs just before eyelid separation in rats, preparing the occipital cortex to patterned vision [38]. We observed a similar phenomenon here. Before 34 PMW, low-volume “clicks”, near the background noise of neonatal ward, evoke cortical responses with power spectrum and topographic characteristics similar to those evoked by voice stimuli at conversational volume. We show that from 35 PMW on, a rapid elimination of auditory-evoked delta-brushes on temporal electrodes allows the expression of more mature auditory evoked responses having smaller amplitude and more diffuse localization. We therefore hypothesize a similar preparation of the auditory cortex for birth that consists of changing from an *in utero* mode which compensates for attenuated sounds by burst amplification, to the extra-uterine hearing mode which requires adaptation to new, non-filtered, acoustic environment.

The difference between cortical responses to non-verbal and verbal stimuli was detectable from 34 PMW in QS: response to “click” became diffuse whereas response to voice remained limited to temporal electrodes. Our findings are in accordance with studies of the effect of stimulus type on CAEPs in full-term newborn which showed that “click”-evoked CAEPs are maximal at central electrodes (N1 and P2 component), while speech-evoked CAEPs show a range of topographical patterns including immature response similar to that seen in preterm infant [45], [65]. These topographic differences might reflect emerging tonotopic organization, the generators coding for phonetic changes being more posterior and dorsal than those coding for similar acoustic changes without any linguistic value [45], [66]. However, these changes do not explain why the diffuse “click” response is observed only in QS, and not in AS. In the premature infant Monod and Garma showed that after 33–34 PMW QS starts inducing different reactivity to auditory stimuli (clicks or tone bursts) for CAEP and for motor reactivity as compare to AS (increase of the amplitude of N2 component of CAEP, decrease of startles, limbs jerks, eyelids blinks) [67]. The N2 component of CAEP in children and adults is known to be enhanced by the first stages of non-rapid eye movement (NREM) sleep (equivalent of QS in preterm) [68]. Moreover, in children and adults, abrupt, and rare sensory stimuli whatever the modality are known to evoke vertex sharp waves and K-complexes in frontal-central areas in the first stages of NREM sleep [69], [70]. K-complexes are supposed to reflect inhibitory mechanisms associated with impaired transmission of sensory influx likely protecting QS from arousal [67]–[70]. All together these data suggest the emergence of some sleep-promoting mechanism of QS from 34 PMW and we may speculate that non familiar, abrupt stimuli such as “click” induce diffuse response whereas voice does not. In our study auditory stimuli presented during the hypoactive periods of EEG in QS triggered evoked auditory responses more efficiently than stimuli presented randomly. We therefore recommend stimulating during hypoactive periods of EEG in QS to evoke temporal delta-brushes by auditory stimuli.

How the differences in auditory experience *in utero* versus *ex utero* might impact the development of the auditory system is unknown. *In utero,* fetal behavioral responses are first observed with low frequencies, those that predominate in fetal natural acoustic environment and for speech (higher frequencies being attenuated up to 40 dB) [50]. The fetal noise floor consists of continuous cardiovascular, respiratory, intestinal sounds, maternal body movements as well as vocalizations and externally generated sounds [71]. *Ex utero*, prematurely born neonates are exposed to all types of frequencies and intensities of non-filtered air born sounds as well as unfiltered speech. In our neonatal department the background noise was 50–53 dB (SPL), similar to levels reported in NICU in previous studies [52]. Using event related potentials, a recent study showed that the premature infant does not take advantage of his richer, unfiltered exposure to speech to accelerate speech acquisition. The expected decrease in amplitude of the mismatch response to a nonnative change of phoneme at the end of the first year of life was dependent on maturational age and not on the duration of exposure to broadcast speech [72]. Early exposure studies with both avian and mammalian species have highlighted the importance of normal patterns (in terms of the type and amount of sensory stimulation), and suggests that when the whole amount of stimulation exceeds a certain threshold, prenatal auditory learning capacity can be compromised [73]. Our data confirm the important role of external sounds to stimulate the fetal auditory cortex: even a low intensity “click” was able to evoke delta-brushes in the temporal cortex before 35 PMW. From this perspective we can hypothesize that over-stimulation with inappropriate frequencies and intensity sounds before 35 PMW could have deleterious effects on auditory cortex maturation of prematurely born infants.

## Supporting Information

Table S1 **Base power spectrum of EEG in global frequency band (0.5–31.5 Hz) according to age expressed in postmenstrual weeks (PMW) of premature infants and type of sleep in a “periodic population”**
(DOCX)

Table S2 **Significant EEG power increase rate after auditory stimuli in the 32–33 postmenstrual weeks age group in quiet sleep.**
(DOCX)

Table S3 **Significant EEG power increase rate after auditory stimuli in the 32–33 postmenstrual weeks age group in active sleep.**
(DOCX)

Table S4 **Significant EEG power increase rate after auditory stimuli in the 34–35 postmenstrual weeks age group in quiet sleep.**
(DOCX)

Table S5 **Significant EEG power increase rate after auditory stimuli in the 34–35 postmenstrual weeks age group in active sleep.**
(DOCX)

Table S6 **Significant EEG power increase rate after auditory stimuli in the 36–37 postmenstrual weeks age group in quiet sleep.**
(DOCX)

Table S7 **Significant EEG power increase rate after auditory stimuli in the 36–37 postmenstrual weeks age group in active sleep.**
(DOCX)
