# Supplementary material for: Auditory Stimuli Mimicking Ambient Sounds Drive Temporal “Delta-Brushes” in Premature Infants
Source: PLoS One. 2013 Nov 11;8(11):e79028. doi: 10.1371/journal.pone.0079028 (PMC3823968; doi:10.1371/journal.pone.0079028)
Supplement: Table S1 — Base power spectrum of EEG in global frequency band (0.5–31.5 Hz) according to age expressed in postmenstrual weeks (PMW) of premature infants and type of sleep in a “periodic population” (DOCX) [file pone.0079028.s001.docx]

**Table S 1: Base power spectrum of EEG in global frequency band (0.5-31.5 Hz) according to age expressed in postmenstrual weeks (PMW) of premature infants and type of sleep in a “periodic population”**

| **Electrode**  **Frequency band (Hz)** | **Quiet sleep Mean ± standard-error** | | **p-value** | **Active sleep Mean ± standard-error** | | **p-value** |
| --- | --- | --- | --- | --- | --- | --- |
|  |  |  |  |  |  |  |
|  | **32-33 PMW** | **34-37 PMW** |  | **32-33 PMW** | **34-37 PMW** |  |
|  |  |  |  |  |  |  |
| **C3, 0.5-31.5** | 977.6 ± 1537.7 | 469.4 ± 1367.7 | **<.001** | 774.2 ± 1134.8 | 356.2 ± 568.9 | **<.001** |
| **C4, 0.5-31.5** | 968.3 ± 1931.8 | 457.4 ± 1044.2 | **<.001** | 721.3 ± 1077.4 | 435.0 ± 1259.0 | **<.001** |
| **CZ, 0.5-31.5** | 735.6 ± 823.8 | 583.7 ± 1758.5 | **<.001** | 734.4 ± 684.1 | 564.4 ± 1395.9 | **<.001** |
| **FP1, 0.5-31.5** | 688.4 ± 811.7 | 396.6 ± 566.6 | **<.001** | 763.3 ± 862.5 | 588.2 ± 2072.3 | **<.001** |
| **FP2, 0.5-31.5** | 1140.3 ± 3483.6 | 401.5 ± 623.5 | **<.001** | 824.1 ± 888.7 | 457.4 ± 475.2 | **<.001** |
| **O1, 0.5-31.5** | 1132.5 ± 2015.6 | 559.3 ± 872.7 | **<.001** | 1331.2 ± 1905.0 | 759.5 ± 1433.7 | **<.001** |
| **O2, 0.5-31.5** | 1147.0 ± 2363.7 | 686.7 ± 1105.2 | **0.068** | 1424.2 ± 2426.3 | 679.2 ± 863.7 | **<.001** |
| **T3, 0.5-31.5** | 1342.5 ± 2104.0 | 650.8 ± 1153.5 | **<.001** | 847.2 ± 901.7 | 707.1 ± 2498.8 | **<.001** |
| **T4, 0.5-31.5** | 1008.9 ± 1921.4 | 482.4 ± 668.8 | **<.001** | 683.3 ± 726.0 | 476.8 ± 836.9 | **<.001** |
| **T5, 0.5-31.5** | 1197.0 ± 2155.9 | 454.8 ± 716.6 | **<.001** | 1086.4 ± 1519.8 | 601.9 ± 1661.7 | **<.001** |
| **T6, 0.5-31.5** | 1423.7 ± 2620.1 | 575.1 ± 1050.7 | **<.001** | 998.3 ± 1553.2 | 535.6 ± 1148.4 | **<.001** |
