# Supplementary material for: Auditory Stimuli Mimicking Ambient Sounds Drive Temporal “Delta-Brushes” in Premature Infants
Source: PLoS One. 2013 Nov 11;8(11):e79028. doi: 10.1371/journal.pone.0079028 (PMC3823968; doi:10.1371/journal.pone.0079028)
Supplement: Table S2 — Significant EEG power increase rate after auditory stimuli in the 32–33 postmenstrual weeks age group in quiet sleep. (DOCX) [file pone.0079028.s002.docx]

**Table S2**: **Significant EEG power increase rate after auditory stimuli in 32-33 postmenstrual weeks age group in quiet sleep**.

| **Electrode** | **Stimulus “click”** | | **Stimulus “voice”** | | **Difference “click”-“voice”** |
| --- | --- | --- | --- | --- | --- |
| **Frequency band (Hz)** | **Effect** | **P-value** | **Effect** | **P-value** | **p-value (interaction)** |
| **CZ, 1-3.5** | 1.51 | **0.0004** | 1.20 | 0.12 | 0.19 |
| **CZ, 7.5-13** | 1.30 | **0.01** | 1.11 | 0.31 | 0.34 |
| **T3, 13.5-31.5** | 2.02 | **0.01** | **1.75** | **0.006** | 0.67 |
| **T3, 1-3.5** | 2.93 | **0.0006** | **2.12** | **0.002** | 0.41 |
| **T3, 4-7** | 2.51 | **0.003** | **1.94** | **0.002** | 0.48 |
| **T3, 7.5-13** | 3.59 | **0.0002** | **1.80** | **0.01** | 0.08 |
| **T4, 13.5-31.5** | 2.05 | **0.01** | **2.33** | **<.0001** | 0.71 |
| **T4, 1-3.5** | 2.86 | **0.001** | **2.54** | **<.0001** | 0.76 |
| **T4, 4-7** | 2.43 | **0.004** | **2.41** | **<.0001** | 0.99 |
| **T4, 7.5-13** | 2.43 | **0.006** | **2.45** | **0.0001** | 0.99 |
| **T5, 1-3.5** | 2.97 | **0.002** | 1.21 | 0.45 | 0.04 |
| **T6, 0.5-31.5** | 2.58 | **0.007** | 1.33 | 0.26 | 0.12 |
| **T6, 1-3.5** | 2.67 | **0.005** | 1.41 | 0.19 | 0.14 |
