# Supplementary material for: Auditory Stimuli Mimicking Ambient Sounds Drive Temporal “Delta-Brushes” in Premature Infants
Source: PLoS One. 2013 Nov 11;8(11):e79028. doi: 10.1371/journal.pone.0079028 (PMC3823968; doi:10.1371/journal.pone.0079028)
Supplement: Table S3 — Significant EEG power increase rate after auditory stimuli in the 32–33 postmenstrual weeks age group in active sleep. (DOCX) [file pone.0079028.s003.docx]

Table S3: Significant EEG power increase rate after auditory stimuli in 32-33 PMW postmenstrual weeks age group in active sleep.

| **Electrode** | **Stimulus “click”** | | **Stimulus “voice”** | | **Difference “click”-”voice”** |
| --- | --- | --- | --- | --- | --- |
| **Frequency band (Hz)** | **Effect** | **P-value** | **Effect** | **P-value** | **p-value (interaction)** |
| **C3, 1-3.5** | **1.54** | **0.01** | 1.09 | 0.60 | 0.14 |
| **C3, 7.5-13** | **1.60** | **0.002** | 1.18 | 0.30 | 0.16 |
| **T3, 13.5-31** | **1.80** | **0.0003** | **1.59** | **0.005** | 0.60 |
| **T3, 1-37.5-13** | **1.75** | **0.002** | 1.37 | 0.07 | 0.32 |
| **T3, 4-7** | **1.96** | **<.0001** | 1.35 | 0.06 | 0.10 |
| **T3, 7.5-13** | **2.18** | **<.0001** | **1.75** | **0.0004** | 0.33 |
| **T4, 7.5-13** | **1.54** | **0.004** | **1.50** | **0.004** | 0.90 |
