# Supplementary material for: Auditory Stimuli Mimicking Ambient Sounds Drive Temporal “Delta-Brushes” in Premature Infants
Source: PLoS One. 2013 Nov 11;8(11):e79028. doi: 10.1371/journal.pone.0079028 (PMC3823968; doi:10.1371/journal.pone.0079028)
Supplement: Table S4 — Significant EEG power increase rate after auditory stimuli in the 34–35 postmenstrual weeks age group in quiet sleep. (DOCX) [file pone.0079028.s004.docx]

Table S4: Significant EEG power increase rate after auditory stimuli in 34-35 postmenstrual weeks age group in quiet sleep.

| **Electrode** | **Stimulus “click”** | | **Stimulus “voice”** | | **Difference “click”-“voice”** |
| --- | --- | --- | --- | --- | --- |
| **Frequency band (Hz)** | **Effect** | **P-value** | **Effect** | **P-value** | **p-value (interaction)** |
| **CZ, 1-3.5** | **2.10** | **0.0002** | 0.93 | 0.72 | **0.0026** |
| **C3, 7.5-13** | **2.06** | **0.005** | 1.19 | 0.29 | 0.0738 |
| **C4, 4-7** | **1.61** | **0.001** | 1.15 | 0.39 | 0.1295 |
| **FP1,** 1-3.5 | **1.87** | **0.002** | 0.73 | 0.10 | **0.0002** |
| **FP2, 13.5-31** | **1.49** | **0.0009** | 1.05 | 0.72 | 0.0737 |
| **FP2, 1-3.5** | **2.15** | **<.0001** | 0.90 | 0.60 | **0.0004** |
| **FP2, 4-7** | **1.46** | **0.0047** | 1.09 | 0.58 | 0.1862 |
| **FP2, 7.5-13** | **1.87** | **<.0001** | 1.06 | 0.70 | 0.0116 |
| **O1, 1-3.5** | **1.64** | **0.0008** | 1.01 | 0.94 | 0.0396 |
| **O1, 7.5-13** | **1.64** | **0.0006** | 1.07 | 0.68 | 0.0604 |
| **O2, 13.5-31** | **1.59** | **0.0004** | 0.84 | 0.29 | **0.0020** |
| **O2, 1-3.5** | **1.79** | **0.0009** | 0.81 | 0.32 | **0.0038** |
| **O2, 4-7** | **1.68** | **0.0006** | 0.85 | 0.41 | **0.0051** |
| **O2, 7.5-13** | **1.89** | **<.0001** | 0.78 | 0.19 | **0.0003** |
| **T3, 1-3.5** | **2.00** | **0.0002** | 1.18 | 0.38 | 0.0440 |
| **T4, 13.5-31** | **1.85** | **<.0001** | **1.57** | **0.005** | 0.4286 |
| **T4, 1-3.5** | **2.79** | **<.0001** | **1.69** | **0.005** | 0.0411 |
| **T4, 4-7** | **1.98** | **<.0001** | **1.69** | **0.002** | 0.4886 |
| **T4, 7.5-13** | **2.18** | **<.0001** | **1.74** | **0.002** | 0.3183 |
| **T5, 1-3.5** | **1.99** | **<.0001** | 1.15 | 0.50 | 0.0366 |
