# Supplementary material for: Auditory Stimuli Mimicking Ambient Sounds Drive Temporal “Delta-Brushes” in Premature Infants
Source: PLoS One. 2013 Nov 11;8(11):e79028. doi: 10.1371/journal.pone.0079028 (PMC3823968; doi:10.1371/journal.pone.0079028)
Supplement: Table S5 — Significant EEG power increase rate after auditory stimuli in the 34–35 postmenstrual weeks age group in active sleep. (DOCX) [file pone.0079028.s005.docx]

**Table S5: Significant EEG power increase rate after auditory stimuli in 34-35 postmenstrual weeks age group in active sleep.**

| **Electrode** | **Stimulus “click”** | | **Stimulus “voice”** | | **Difference “click”-“voice”** |
| --- | --- | --- | --- | --- | --- |
| **Frequency band (Hz)** | **Effect** | **P-value** | **Effect** | **P-value** | **p-value (interaction)** |
| **T4, 0.5-31.5** | **2.31** | **<.0001** | 1.46 | 0.615 | 0.10 |
| **T4, 13,5-31** | **2.39** | **<.0001** | 1.18 | 0.3410 | 0.01 |
| **T4, 7.5-13** | **2.35** | **<.0001** | 1.31 | 0.1152 | 0.02 |
| **T4, 1-3.5** | **2.52** | **<.0001** | 1.30 | 0.1595 | 0.02 |
| **T4, 4-7.5** | **1.80** | **0.0018** | 1.25 | 0.1739 | 0.14 |
