# Supplementary material for: Auditory Stimuli Mimicking Ambient Sounds Drive Temporal “Delta-Brushes” in Premature Infants
Source: PLoS One. 2013 Nov 11;8(11):e79028. doi: 10.1371/journal.pone.0079028 (PMC3823968; doi:10.1371/journal.pone.0079028)
Supplement: Table S6 — Significant EEG power increase rate after auditory stimuli in the 36–37 postmenstrual weeks age group in quiet sleep. (DOCX) [file pone.0079028.s006.docx]

**Table S6: Significant EEG power increase rate after auditory stimuli in 36-37 postmenstrual weeks age group in quiet sleep.**

| **Electrode** | **Stimulus “click”** | | **Stimulus “voice”** | | **Difference “click”-“voice”** |
| --- | --- | --- | --- | --- | --- |
| **Frequency band (Hz)** | **Effect** | **P-value** | **Effect** | **P-value** | **p-value ( interaction)** |
| **FP2, 1-3.5** | **1.56** | **0.0055** | 1.04 | 0.7449 | 0.0639 |
| **O1, 1-3.5** | **1.58** | **0.0025** | 0.91 | 0.5661 | **0.0101** |
| **T4, 1-3.5** | **1.64** | **0 .0022** | 1.32 | 0.0493 | 0.3229 |
| **T4, 7.5-13** | **1.59** | **0.0013** | **1.42** | **0.0084** | 0.5649 |
| **T4, 4-7.5** | **1.46** | **0.0055** | **1.44** | **0.0040** | 0.9532 |
| **T6, 1-3.5** | **1.48** | **0.0094** | 0.98 | 0.9453 | 0.0600 |
