# Supplementary material for: Auditory Stimuli Mimicking Ambient Sounds Drive Temporal “Delta-Brushes” in Premature Infants
Source: PLoS One. 2013 Nov 11;8(11):e79028. doi: 10.1371/journal.pone.0079028 (PMC3823968; doi:10.1371/journal.pone.0079028)
Supplement: Table S7 — Significant EEG power increase rate after auditory stimuli in the 36–37 postmenstrual weeks age group in active sleep. (DOCX) [file pone.0079028.s007.docx]

Table S7: Significant EEG power increase rate after auditory stimuli in 36-37 postmenstrual weeks age group in active sleep.

| **Electrode** | **Stimulus “click”** | | **Stimulus “voice”** | | **Difference “click”-“voice”** |
| --- | --- | --- | --- | --- | --- |
| **Frequency band (Hz)** | **Effect** | **P-value** | **Effect** | **P-value** | **p-value (interaction)** |
| **T3, 13.5-31** | **1.22** | **0.0105** | 1.11 | 0.1904 | 0.44 |
| **T3, 1-3.5** | **1.34** | **0.0017** | 0.93 | 0.4350 | 0.008 |
| **T3, 4-7** | **1.29** | **0.0017** | 0.99 | 0.9459 | 0.03 |
| **T3, 7.5-13** | **1.28** | **0.0013** | 1.05 | 0.5072 | 0.08 |
| **T4, 7.5-13** | **1.22** | **0.0053** | **1.25** | **0.0021** | 0.81 |
